# Evaluation of the E-PRE-DELIRIC prediction model for ICU delirium: a retrospective validation in a UK general ICU

**DOI:** 10.1186/s13054-020-2838-2

**Published:** 2020-03-30

**Authors:** Sarah L. Cowan, Jacobus Preller, Robert J. B. Goudie

**Affiliations:** 1 Addenbrooke’s Hospital, Cambridge, CB2 0QQ UK; 2 MRC Biostatistics Unit, School of Clinical Medicine, University of Cambridge, Cambridge, CB2 0SR UK

**Keywords:** Clinical scoring systems, Critical care, Delirium, Intensive care, Prediction model, Validation

## Introduction

E-PRE-DELIRIC is a point-of-admission ICU delirium risk prediction tool [1], with reported good or moderate performance [2–4]. In this study, we assessed its performance in a large UK teaching hospital general ICU using routinely collected data, as approved by the local Research Data Governance Committee.

## Methods

We retrospectively analysed data for 2445 consecutive ICU admissions (November 2014 to June 2017). Patients were routinely assessed for delirium, using twice daily Confusion Assessment Method for the ICU (CAM-ICU) assessment [5]. As in previous E-PRE-DELIRIC studies [1–4], delirium was defined as any positive CAM-ICU assessment or antipsychotic initiation while on ICU.

We adopted the original E-PRE-DELIRIC exclusion criteria [1], excluding 683 ICU admissions for ICU stay < 24 h (425 admissions), incomplete CAM-ICU data (152), delirium on admission (50), comatose throughout entire ICU stay (47), and age under 18 (9). Sixteen admissions were excluded due to missing E-PRE-DELIRIC components; 1746 admissions (1569 unique patients) remained for analysis; this 71.4% inclusion rate is consistent with previous studies (Table 1).

**Table 1:** Patient characteristics in this study, the E-PRE-DELIRIC development dataset [1] and other validation studies [2–4]

Factor	This study	Development dataset [1]	DECISION study [2, 3]	Green et al. [4]
Admissions during study period, *n*	2445	–	2802	803
Included in analysis, *n* (%)	1746 (71.4)	1962 (–)	2178 (77.7)	455 (56.7)
Delirium, *n* (%)	763 (43.7)	481 (24.5)	466 (21.4)	160 (35.2)
Age (years), mean (Q1–Q3, min/max)	58.6 (47.0–71.8, 18/94)	61.7 (53–74, 18/95)	62.1 (–)	66.7 (49.0–77.3, –/–)
Male, *n* (%)	1010 (57.8)	1166 (59.4)	1324 (60.8)	241 (53.0)
Admission category, *n* (%)				
Surgery	813 (46.6)	1019 (51.9)	1079 (49.5)	–
Medicine	837 (47.9)	683 (34.8)	859 (39.3)	–
Trauma	33 (1.9)	90 (4.6)	86 (4.0)	–
Neurology/neurosurgery	63 (3.6)	170 (8.7)	157 (7.2)	–
Urgent admission, *n* (%)	1534 (87.9)	1163 (59.3)	1345 (61.8)	–
APACHE II	20.0 (mean)	–	17.4 (mean)	16 (median)
ICU LoS (days), median (Q1–Q3, min/max)	4.5 (2.4–10.0, 1.0/184.0)	2.0 (1–6, 1/133)	3.0 (2–6, 1/96)	2.6 (1.5–4.4, –/–)
ICU mortality, *n* (%)	210 (12.0)	–	–	17 (3.7)

– indicates the figure was not reported

## Results and discussion

Seven hundred sixty-three delirium cases were identified (43.7% of ICU admissions), a higher incidence than reported previously (Table 1). This is likely due to differences in the study population compared to previous studies: more patients were classified as urgent, the mean APACHE II score was higher, and median length of stay (LoS) was longer (Table 1).

**Fig. 1:**
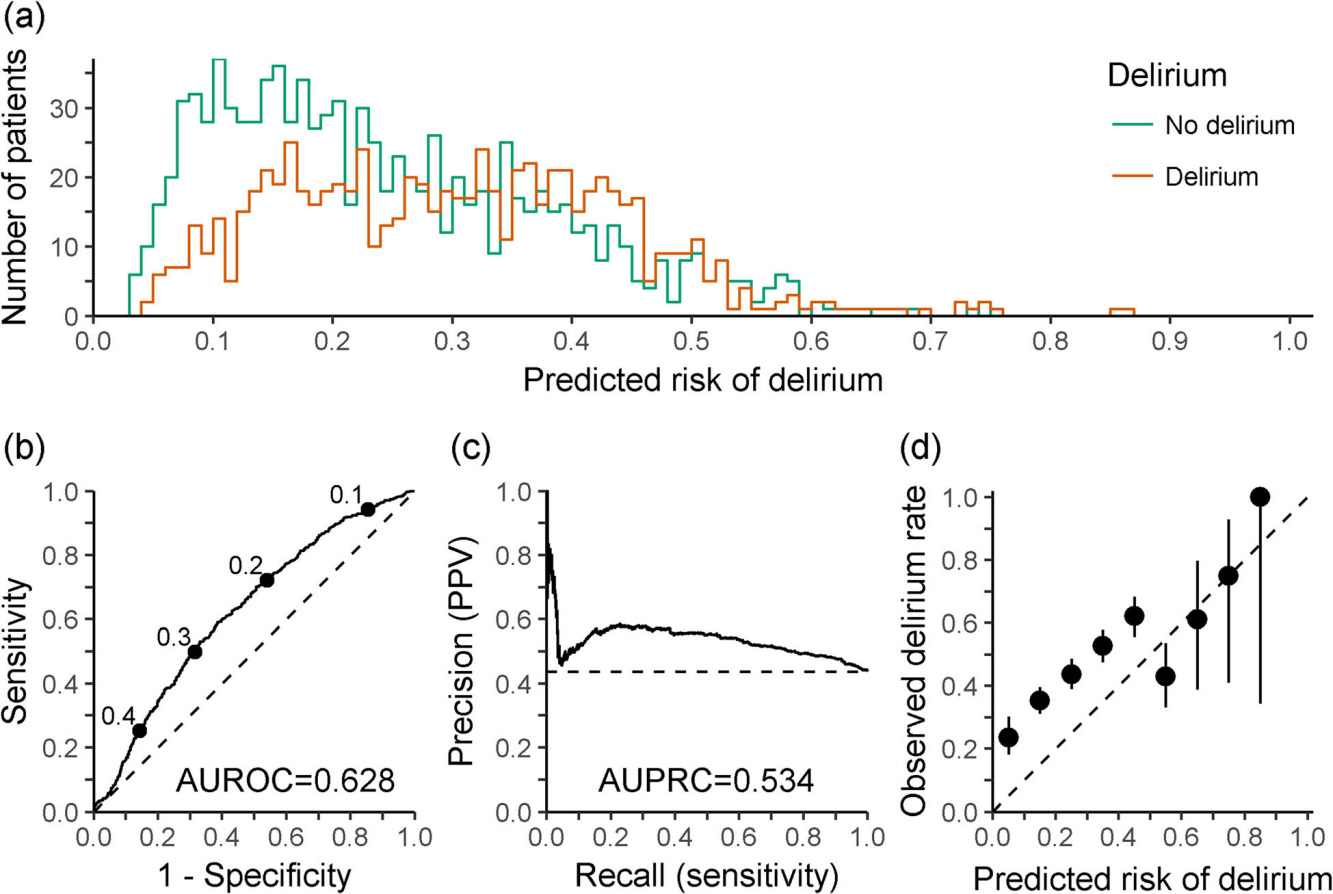
**a** Histogram of predicted risk of delirium by delirium status. **b** Receiver operator characteristic plot, with labels indicating the corresponding threshold and the dashed line indicating the line of no discrimination. **c** Precision-recall plot, with the 43.7% observed incidence indicated by the dashed line. **d** Calibration plot (with 95% CI), by tenths of predicted risk, with the dashed line indicating perfect calibration

The mean E-PRE-DELIRIC score was 0.269 (Q1–Q3; 0.154–0.371). The histogram of E-PRE-DELIRIC scores shows extensive overlap between patients who did and did not develop delirium (Fig. 1a). The receiver operator characteristic (ROC) curve (Fig. 1b) and the precision-recall (PR) curve (Fig. 1c), showing precision (positive predictive value (PPV)) against recall (sensitivity), both indicate moderate-to-poor discriminative performance. The area under the ROC (AUROC) was 0.628 (95% CI 0.602–0.653). The area under the PR curve (AUPRC) was 0.534. For sensitivity > 0.1, PPV was between 0.437 and 0.585, indicating only around half of the patients predicted to develop delirium actually did, in a population with 43.7% incidence. Refitting the E-PRE-DELIRIC logistic regression model to our data hardly improved discrimination: AUROC was 0.648 (95% CI 0.622–0.673) and AUPRC was 0.566.

The calibration plot, of predicted risk against observed delirium rate, shows the risk of delirium is considerably underestimated, especially in patients with predicted risk of delirium less than 0.5 (Fig. 1d). Poor calibration is corroborated by the calibration slope model logit(probability of delirium) = alpha + beta ×logit(*p*), where *p* is the E-PRE-DELIRIC score [6]. The estimated slope beta = 0.58 (95% CI 0.46–0.71) is significantly below 1, indicating the predicted probabilities are overly variable; and the estimated intercept alpha = 0.84 (95% CI 0.74–0.95) is significantly above 0 when fixing beta = 1, indicating the predicted probabilities are predominantly too low. E-PRE-DELIRIC is particularly poorly calibrated for the surgical patients in the study, many of whom have major intraabdominal pathology: those with predicted risk < 10% had an observed incidence of 26%.

Of 763 delirium cases, 563 were CAM-ICU positive and 200 were included due to antipsychotic initiation. When including only CAM-ICU-positive delirium, calibration was improved (alpha = 0.29) but remained overly variable (beta = 0.52), while discrimination was similar (AUROC 0.615; AUPRC 0.396, with 32.2% observed incidence).

While E-PRE-DELIRIC is intended as a point-of-admission score, some of its exclusion criteria are retrospective (LoS; CAM-ICU completeness; comatose throughout). To assess real-world performance, we repeated our analysis without these criteria. The AUROC (0.615) and AUPRC (0.423, with 35.0% observed incidence) remained similar.

## Conclusion

In this population, the E-PRE-DELIRIC score is not as discriminative or as well calibrated as previously reported. PPV was only slightly higher than delirium incidence, meaning the utility of E-PRE-DELIRIC for guiding clinical decision-making in this population is limited.

## Data Availability

The data that support the findings of this study are available from Cambridge Clinical Informatics, but restrictions apply to the availability of these data, which were used under license for the current study, and so are not publicly available. The data are anonymised, but to preserve patient confidentiality and privacy, the Data Use Agreement states that the data cannot be deposited into open access repositories of any kind. Anyone wishing to access data must submit and receive approval for access to these data from the Cambridge Clinical Informatics Research Data Governance Committee.

## Abbreviations

AUROC: Area under the ROC curve
AUPRC: Area under the PR curve
CAM-ICU: Confusion Assessment Method for the ICU
E-PRE-DELIRIC: Early prediction model for delirium in ICU patients
ICU: Intensive care unit
LoS: Length of stay
PPV: Positive predictive value
PR: Precision-recall
ROC: Receiver operator characteristic
